# Proposal of an objective formula-based model for equitable ranking of veterinary colleges

**DOI:** 10.3389/fvets.2025.1526980

**Published:** 2025-02-05

**Authors:** Robert M. Gogal, Steven David Holladay

**Affiliations:** Department of Biomedical Sciences, College of Veterinary Medicine, University of Georgia, Athens, GA, United States

**Keywords:** college, university, rankings, formula-based, model

## Abstract

**Introduction:**

We suggest an objective ranking formula that has wide national and international application. The new formula is presented with U.S. Colleges of Veterinary Medicine as an example. Methods used by ranking agencies differ, as do the ranks they generate. In some instances, relatively narrow information is collected from a limited number of polled contributors, raising questions about strength and validity of the generated ranks.

**Methods:**

A new formula-based weighted model for ranking of veterinary colleges is proposed. Numbers fed into the formula parameters, for instance teaching hospital case load, faculty number and research expenditures, derive from real and measurable qualities of each college and drive an objective final ranking score for each veterinary college. This formula is designed to be readily modified within and beyond the veterinary profession.

**Results:**

The new ranking system works from calculations that are straightforward and transparent with little room for subjective interpretation. It is designed to provide a rigorous and defendable institutional rank for all veterinary colleges.

**Discussion:**

The new ranking system is designed to be readily adaptable within veterinary colleges as the profession changes, as well as to academic institutions with focuses other than veterinary medicine. The goal is to provide a new model that is useful for objective comparisons.

## Introduction

In the academic world, an area that is consistently under debate is the methods used to rank colleges and universities at the national and international level (1, 2). This is not surprising as an academic institution’s rank significantly impacts prospective undergraduate, graduate and professional student application decision making, private donor and alumni fundraising success, and ability to attract and hire talented faculty and staff (3). Major criticisms by academic institutions with the current ranking approaches include a high degree of subjectivity by some procedures, unsubstantiated or weak metrics, editorial overreach, a lack of transparency in the data analyzed for generating rankings, and inconsistent year-to-year fluctuations in institutional ranks. For instance, university rankings by the Times Higher Education Ranking (THE) and the Shanghai Jiao Tong University’s Academic Ranking of World Universities (ARWU) showed mean year-to-year fluctuation increases of up to 60% in groups of universities aggregated by lower rank (1). The latter authors suggested that the THE and ARWU ranking systems might alter their procedures to have lower-ranked universities summarized only in groups of 25 or 50. As a result of such potential challenges in ranking systems, some academic institutions choose to refrain from participating in ranking surveys or data submission, which decreases utility of the rankings (4). This report uses U.S. veterinary colleges to present a novel and objective ranking metric formula designed to minimize year-to-year fluctuations and improve objectivity of rankings of educational institutions.

As of 2024, there are approximately 24,724 universities and colleges worldwide of which there are estimated 3,900 medical schools (*n* = 173 in the U.S.) and an estimated 550 veterinary colleges/schools (5). Of these, 34 of 49 American Veterinary Medical Association (AVMA) accredited veterinary colleges are in the United States, which represents 6.0% of all worldwide veterinary colleges. In the United States, it is required that each veterinary college attain and retain AVMA accreditation in order for graduates to sit for the national boards and obtain licensing to practice veterinary medicine. To a prospective pre-veterinary student, this requirement may be sufficient alone when selecting a veterinary college to attend. However, U.S. veterinary colleges vary significantly in the type of programs (clinical and research) as well as facilities (i.e., on-site hospitals) that they offer.

There are several independent entities that rank U.S. veterinary colleges based on different parameters of programs and facilities and publish their outcomes online. Of these, the U.S. News & World Report ranking is most recognized followed by those of Quacquarelli Symonds (QS) and the Academic Ranking of World Universities (6). It might be viewed as noteworthy that these three widely followed agencies differ significantly in the rubrics, they use for ranking veterinary colleges.

The U.S. News & World Report acquires their list of U.S. veterinary colleges from the American Veterinary Medical Association (AVMA), which is responsible for the accreditation process. Only U.S. veterinary colleges with full accreditation are then ranked by this agency. One advantage of this ranking system to an individual residing in the United States is therefore that it compares only U.S. veterinary colleges. The U.S. News & World Report sends a survey to two administrators at each of veterinary college, results of which are used to rank the schools. These individuals are typically the dean of the college and one other senior administrator (i.e., hospital director or an associate dean). The participants are asked to complete a one-question peer assessment survey in which they are tasked with assessing the academic quality of the programs for each of their peer institutions. This rating is on a scale of 1–5 where 1 is considered marginal and 5 is outstanding. The most recent peer assessment cycle was conducted in Fall of 2022 through the early part of 2023 with 53% of contacted veterinary college administrators responding to the survey (7). Concern has been expressed that low administrator participation rate may challenge the validity of this ranking system, however, the U.S. News & World Report Veterinary School Rankings remains as the primary reference used by pre-veterinary students and private entities when selecting a college to attend or with which to collaborate (8).

The QS World University Rankings: Veterinary Science, as the name implies, also ranks international veterinary schools. In 2020, QS World University Rankings: Veterinary Science were based on 3 indicators: academic reputation, citations per manuscript published by college faculty members, and H-index citations of these manuscripts to compute an overall score. The most recent survey from 2023 expanded to five indicators: academic reputation, employer reputation, research citations per paper, citation H-index and international research network (9). For both the 2020 and 2023 rankings, academics and graduate employers worldwide were surveyed. In the 2023 QS World University Rankings, >130,000 academics and > 75,000 graduate employers were surveyed, not for all 5 indicators but for those of relevance to the surveyee (9). It was at times not clear if a veterinary college was ranked independent of the parent university or whether the ranking was partly influenced by the university’s international reputation. This is an advantage of the U.S. News & World Report which does rank the universities and professional colleges independently.

ThoughtCo. published an online article in September of 2020 titled, “Top 10 Best Veterinary Schools in the U.S.” authored by Dr. Allen Grove who is professor of English at Alfred University, New York (10). Although well written, the author provided limited information as to why these veterinary schools were ranked as his top 10. In some summaries, he also cited U.S. News & World Report as part of his ranking basis. Many of Dr. Grove’s top 10 are no longer in the top 10 based on the 2023 the U.S. News World & Report.

EduRank.org is another independent ranking organization which strives to collect data independent of the institutions and apply a metric-based scoring system to three categories: institutional research performance (45%), non-academic prominence (45%), and alumni score (10%) (11). In their 2018 online posting, institutional research performance was determined using the OpenAlex database (OurResearch, Sanford, NC; ARCADIA, London, UK) to collect scientific publications and citation number and employ a calculation to weight the data. Non-academic prominence was determined using search engines (i.e., Google) to screen individual web page-backlinks to a specific institution via other sites as an indicator of institutional status. The alumni score was based on the number of page views an institution’s graduates and affiliates made on Wikipedia’s 43 language versions (11). A perceived benefit of this ranking is that the data were independently collected, and the authors employed a metric approach to their ranking.

Accredited Schools Online (ASO) is another private independent organization that ranks U.S. veterinary colleges. They employ a metric-based system focused on an institution’s affordability, academic quality, reputation, and program offerings (12). They define affordability as a college’s “net price” as determined by the average number of student grants and scholarships awarded as well as the median student debt post-graduation. Academic quality was determined by the student-faculty ratio, student retention, and graduation rate. The college’s reputation was assessed by evaluating the admission and enrollment rates as well as average post-graduate earnings. Program offerings looked at the percentage of degrees offered plus the number of students enrolled in online courses. A review of the ASO 2023 Top U.S. Veterinary Schools and Programs reveals that 5 of these 20 were not veterinary colleges (12). Of the 15 veterinary colleges that were ranked in the top 20, ASO rankings were not comparable to the 2023 U.S. News & World Report rankings, showing different ranking paradigms yield different results.

Given that there are numerous successful veterinary practitioners graduating from all accredited U.S. veterinary colleges, one might also claim that ranking accredited U.S. veterinary colleges is unnecessary. Indeed, if the primary goal of a veterinary college is to produce competent general practitioners, then veterinary college rankings may not be of high need. However, as mentioned earlier, most full-service veterinary colleges with a veterinary teaching hospital offer advanced programs (e.g., clinical internships, clinical residencies, research training and graduate degrees) across a wide field of specialties and these differ by college. Surprisingly, these are often overlooked by the ranking entities. A more valid and defendable approach might be to employ a set of academic and college-based parameters that are readily quantifiable, and easily obtained for each U.S. veterinary college. Resulting data could be inserted into a mathematical formula to generate a purely objective number for mechanistically identical assessment of each veterinary college’s rank.

## Methods

We propose employing a weighted parameter-based formula model for ranking educational institutions. The goal for this weighted-formula approach would be to generate a comprehensive and objective assessment in which, in this example, each U.S. veterinary college is identically evaluated. The rationale for the weighted parameter approach is that some college parameters garner a higher weight while others a lower weight based on past performance indicators. For example, a parameter that would merit a high weight factor would be Hospital Case Load, as this directly impacts a veterinary student’s exposure to clinical cases. Other high weight factor categories would include faculty number (FN), percent of students passing the NAVLE national board examination on 1st attempt over the past 5 years (NAV), graduate student number (GSN: residents, interns, PhD, MS and DVM/PhD) and research (R) defined by total extramural expenditures and research citations. A lower weight factor, but still an important parameter that should contribute to rank, would be tuition plus cost of living, as this predicts total debt associated with obtaining the veterinary medical degree. Adding additional parameters and/or modifying existing ones in this formula-based ranking can readily be done as the profession grows and changes. For non-veterinary colleges, it can be seen that many of these parameters, e.g., tuition; faculty number, directly apply as the formula is adapted to, say, schools of law or to universities offering undergraduate but not graduate degrees.

The ranking formula we propose for veterinary colleges is shown in the top of Table 1 and has seven weighted categories as shown in this table. The categories (W_1_-W_7_) are importance or weight parameters assigned to each category, ranging from 5 (highest weight) to 3 (lower weight). Weights were determined by an email polling of the faculty of the University of Georgia, College of Veterinary Medicine. The faculty were asked to assign a weight from 5 to 3 (most important to lower importance) for each of the formula parameters shown in Table 1. Weight factors shown in Table 1 are means from the faculty polling (*N* = 18 responses), rounded to the nearest whole number. For each score factor, the value assigned derives from college data (e.g., hospital case load) and ranges from 1 to 5 with 5 being the highest possible score. For a formula category with a weight factor of 5 and a score of 5, the numeric return from that portion of the formula would be 25. A category with a weight factor of 4 would have a maximum score of 20 and with a weight factor of 3 would have a maximum score of 15. When all 7 weighted category scores are added together, the maximum attainable score for any college of veterinary medicine would be 145.

**Table 1:** Dual weighted parameter US veterinary college ranking formula.

(T × W_1_) + (FN × W_2_) + (GSN × W_3_) + (HCL × W_4_) + (NAV × W_5_) + (R × W_6_) + (HSF × W_7_) = Rank		
Parameter	Weight factor	Score factor range
T = Tuition, professional students	3	1–5
FN = Faculty number	5	1–5
GSN = Graduate student number	4	1–5
HCL = Hospital case load	5	1–5
NAVLE = % students passing NAVLE, first attempt	4	1–5
R = Research expenditures and program	5	1–5
HSF = Hospital square footage	4	1–5

The score factor for each category is assigned a number between 1 and 5 based on the dataset range for that specific category (Table 2). For example, tuition/costs (T) would include the annual tuition cost, school fees, books/supplies, transportation, personal expenses and cost of living. These cost of living data are available for the United States colleges of veterinary medicine on the internet pages. For some data, for instance the most recent years’ NAVLE pass rate, direct contact with a college’s associate dean of academic affairs may be needed. For the first Table 2 category, five cost-of-living ranges were established based on the T data collected from the 34 U.S. Veterinary colleges. Ranges started with the lowest annual tuition plus cost of living estimate ($37,000) and was increased by the same increment ($7,000) for each employed range except the last. The last range is larger to include the smaller number of more expensive veterinary colleges. The remaining 6 categories were divided into scoring ranges in the same manner.

**Table 2:** Individual category score factor range.

Category (weight)	Scoring basis	Score
Tuition costs (T)	$65,001–$95,076	1
	$58,001–$65,000	2
	$51,001–$58,000	3
	$44,001–$51,000	4
	$37,000–$44,000	5
Faculty number (FN)	<90	1
	90–129	2
	130–169	3
	170–209	4
	>210	5
Graduate student number (GSN)	1–50	1
	51–100	2
	101–150	3
	151–200	4
	201–250+	5
Hospital case load (HCL)	<15,000	1
	15,000–20,000	2
	20,001–25,000	3
	25,001–30,000	4
	>30,000	5
NAVLE pass rate % (NAV)	<81	1
	81.1–85	2
	89.1–93	3
	93.1–97	4
	>97	5
Research rank (R)	1–6	1
	7–12	2
	13–19	3
	20–26	4
	27–34	5
Hospital square feet (HSF)	<50,000	1
	50,000–70,000	2
	70,001–90,000	3
	90,001–110,000	4
	>110,000	5

## Results

To demonstrate how the category (T × W_1_) is scored for each veterinary college, the Year 1 T data from the 34 U.S. colleges are shown in Table 3. The Year 1 T values ranged from $37,390 to $95,076. Cost ranges for scores of 1–5 are then shown in the top of Table 3. Using this approach, the T range for the score of 1 is between $65,001 and $95,076. The T score for a college is then multiplied by the W_1_ score of 4 to attain each college’s tuition cost score factor (T × W_1_). As seen in Tables 3, T × W_1_ ranged from 4 to 20 points awarded for this factor. Other formula categories are then scored in the same manner. An advantage of this formula-based ranking is that the calculations are straightforward and transparent with no room for subjective interpretation. This ranking system is also easily adaptable within veterinary colleges as the profession may change and additional weighted factors are added or removed. The ultimate goal is to facilitate objective and identical data gathering for each veterinary college and provide greater credibility to institutional rankings.

**Table 3:** Tuition cost score factor: US veterinary colleges in-state year 1.

US veterinary colleges (*n* = 34)	Tuition (T) (tuition, fees, supplies, food, housing, personal expenses, transportation)	Category score (T)	Weight score (W_1_)	Tuition cost score factor (T × W_1_)
1	$55,310	3	3	12
2	$71,000	1	3	4
3	$46,634	4	3	16
4	$45,016	4	3	16
5	$75,203	1	3	4
6	$37,390	5	3	20
7	$64,135	2	3	8
8	$46,451	4	3	16
9	$48,616	4	3	16
10	$75,574	1	3	4
11	$54,302	3	3	12
12	$71,421	1	3	4
13	$47,320	4	3	16
14	$46,663	4	3	16
15	$43,668	5	3	20
16	$55,928	3	3	12
17	$40,031	5	3	20
18	$48,046	4	3	16
19	$61,099	2	3	8
20	$63,484	2	3	8
21	$60,319	2	3	8
22	$52,780	3	3	12
23	$67,587	1	3	4
24	$53,687	3	3	12
25	$61,686	2	3	8
26	$57,050	3	3	12
27	$59,560	2	3	8
28	$46,156	4	3	16
29	$61,686	2	3	8
30	$46,986	4	3	16
31	$93,650	1	3	4
32	$95,076	1	3	4
33	$83,800	1	3	4
34	$82,800	1	3	4

Example: Veterinary College × has tuition and other formula category scores as shown in Table 4. These scores derive from publicly available data for the College.

**Table 4:** Rank score for veterinary college X.

Parameter	Category score	Proposed weights
T	4	3
FN	4	5
GSN	3	4
HCL	5	5
NAV	5	4
R	2	5
HSF	3	4

Using the ranking formula:


$$\begin{array}{l} \left(T\times W_{1}\right)+\left(FN\times W_{2}\right)+\left(GSN\times W_{3}\right)+\left(HCL\times W_{4}\right)\\ +\left(NAV\times W_{5}\right)+\left(R\times W_{6}\right)+\left(HSF\times W_{7}\right)=\mathrm{Rank} \end{array}$$

The Rank Score for Veterinary College × is:

(4 × 3) + (4 × 5) + (3 × 4) + (5 × 5) + (5 × 4) + (2 × 5) + (3 × 4) = 111

The maximum rank score the formula can generate would be for presumptive College Y with scores of 5 in each formula category, and would be:

(5 × 3) + (5 × 5) + (5 × 4) + (5 × 5) + (5 × 4) + (5 × 5) + (5 × 4) = 150

The theoretical range for the formula then becomes 0–150. Ranking of all 34 U.S. veterinary colleges using the proposed formula would result in an objective hierarchy of the colleges within this numeric range.

## Discussion

Current methods employed by entities that rank U.S. veterinary colleges differ in the type of subjective and objective data metrics collected and often appear to have noteworthy weaknesses (13–15). It may therefore not be seen as surprising that U.S. veterinary college rankings produced by these entities show many differences. For many administrators, faculty and staff at these veterinary colleges, current methods employed to attain rankings are viewed as subject to question, which may be reflected by poor response rates to data gathering surveys. What is also noteworthy, is that this is not a unique issue to U.S. veterinary colleges but is also a concern expressed by other undergraduate/graduate and professional academic institutions (16–19). Attaining a rigorous and defendable institutional rank would be a clear benefit to a U.S. veterinary college for the reasons already discussed above. The weighted parameter-based formula we present with U.S. veterinary colleges as an example was designed to incorporate key factors of high value to U.S. veterinary colleges, and also to be a readily adjustable model based on peer review and input, as well as able to change in order to meet the evolving needs of the profession. When applying the new formula model for ranking, say, schools of law, new parameters will enter the formula (e.g., size of library collections; realistic moot court competitions) in place of veterinary college parameters not applying to law schools (e.g., hospital case load). With these discipline-specific changes, we suggest the same basic formula model can be widely used to objectively compare diverse categories of educational institutions.

The proposed formula has potential limitations and is designed to be modified as these may be identified. During polling of faculty to determine weight factors used in the formula, several clinical faculty suggested the number of house officers (interns and residents) should be added. We felt this factor was already captured by the HSF and HCL factors and therefore elected at this time to not add house officer number. Weights applied to factors in the proposed formula then all come from a survey of faculty of our College. This survey should be broadened to include members of all U.S. Colleges of Veterinary Medicine, for instance at an upcoming annual meeting of the American Veterinary Medical Association (AVMA). The basic formula should again readily adapt to other disciplines, for instance law schools that may replace “hospital case load” with “number of volumes in the academic law library” or other factors determined by law school experts.

## Data Availability

The raw data supporting the conclusions of this article will be made available by the authors, without undue reservation.
